# Effects of positive social comparative feedback on motor sequence learning and performance expectancies

**DOI:** 10.3389/fpsyg.2022.1005705

**Published:** 2023-01-24

**Authors:** Allison F. Lewis, Rachel Bohnenkamp, Lynn Johnson, Dirk B. den Ouden, Sara Wilcox, Stacy L. Fritz, Jill Campbell Stewart

**Affiliations:** ^1^Department of Exercise Science, University of South Carolina, Columbia, SC, United States; ^2^Department of Communication Sciences and Disorders, University of South Carolina, Columbia, SC, United States

**Keywords:** enhanced expectancies, OPTIMAL theory, motor skill, feedback, kinematic

## Abstract

**Introduction:**

Positive social comparative feedback indicates to the learner that they are performing better than others. While this type feedback supports motor skill learning in some tasks, the effect of social comparative feedback on implicit motor sequence learning remains unknown. The aim of this study was to determine the effect of positive social comparative feedback on the learning of and expectancies for a motor sequence task.

**Methods:**

Forty-eight individuals practiced a joystick-based sequence task and were divided into three feedback groups: CONTROL (no performance feedback), RT ONLY (response time only feedback), and RT+POS (response time plus positive social comparison). Participants attended sessions on two consecutive days: Day 1 for repetitive motor practice/skill acquisition and Day 2 for retention testing. Performance related expectancies, like perceived competence, were measured before and after motor practice on Day 1 and at retention on Day 2.

**Results:**

While all groups improved with practice, the CONTROL group showed better overall performance/learning (faster response times) compared with the RT ONLY group. Despite similar response times, the RT+POS showed higher peak velocities than the RT ONLY group. Overall, the RT+POS and CONTROL demonstrated increases in perceived competence while the RT ONLY group did not.

**Discussion:**

The results of this study suggest that feedback content is an important consideration during motor practice sessions since feedback without context (RT ONLY) may be detrimental to motor sequence learning. The results also suggest that, if providing performance related feedback during practice of a skill that relies on implicit sequence learning processes, comparative context may be necessary for enhancing expectancies and supporting.

## 1. Introduction

Behavioral practice is the foundation of restorative motor rehabilitation and motor skill learning. According to the OPTIMAL (Optimizing Performance Through Intrinsic Motivation and Attention for Learning) theory, positive social comparative feedback during practice (i.e., feedback that indicates to the learner that they are performing better than others) enhances a learner’s expectancies about their performance, thereby benefitting motor performance and learning (Wulf and Lewthwaite, 2016). Positive social comparative feedback is one of several tools that can be applied during practice to enhance expectancies, such as extrinsic reward (Abe et al., 2011; Bacelar et al., 2020), liberal definitions of success (Chiviacowsky et al., 2012; Trempe et al., 2012; Palmer et al., 2016), positive feedback or feedback only after successful trials (Badami et al., 2011; Saemi et al., 2012), and supporting conceptions of ability (Wulf and Lewthwaite, 2009). Expectancies include the learners’ perceived competence, expectations about task outcome (success or failure), and predictions of extrinsic reward (Wulf and Lewthwaite, 2016). In previous research, positive social comparative feedback enhanced learners’ expectancies reflected by improvements in measures of perceived competence, self-efficacy, positive affect, and overall intrinsic motivation toward the motor task (Ávila et al., 2012; Stoate et al., 2012; Wulf et al., 2012, 2014). In addition, learners who received positive social comparative feedback during practice showed better performance and learning at retention testing compared to learners who received performance feedback without positive social comparison (Lewthwaite and Wulf, 2010; Wulf et al., 2012, 2014; Pascua et al., 2015).

Positive social comparative feedback has been shown to support motor skill learning in a range of motor tasks, such as overhead throwing (Pascua et al., 2015), balancing on a stabilometer (Lewthwaite and Wulf, 2010; Wulf et al., 2012; but see Ong and Hodges, 2018), and a sequential timing task (Wulf et al., 2010). Motor skill learning can occur through several different mechanisms and can engage explicit and implicit processes to varying degrees (Spampinato and Celnik, 2021; Leech et al., 2022). Most prior studies support the OPTIMAL theory assumptions showing that expectancy-enhancing practice conditions are beneficial to motor learning (Bacelar et al., 2022). However, some studies challenge this idea showing no benefit (Steel et al., 2016; Ong and Hodges, 2018; Ong et al., 2019; Zobe et al., 2019; Bacelar et al., 2020). Some of these contradictory studies involved tasks that rely on implicit learning processes (Steel et al., 2016; Ong and Hodges, 2018). Thus, it is possible that “optimal” practice conditions may vary based on the motor learning mechanism profile of the task, and further investigation of how expectancy-enhancing practice conditions impact implicit learning is warranted.

Motor sequence learning can occur through both explicit processes, with knowledge of the sequence, and implicit processes, without explicit knowledge or awareness of the sequence (Squire, 1992). Serial target tasks (STTs) have been utilized to study implicit motor sequence learning by embedding random and repeated sequence types into practice (Mang et al., 2016; Baird and Stewart, 2018). This task can provide insight into general sensorimotor learning (random sequence type) and implicit sequence learning (repeated sequence type). Since the sequence order is not provided and the learner is generally unaware of the presence of the sequence, the improvements observed in the repeated sequence performance represent implicit sequence learning. Implicitly learned motor skills have been shown to be more robust and durable in sport situations when a second task or a stressor is introduced (Liao and Masters, 2001; Masters et al., 2008; Lam et al., 2009; Verburgh et al., 2016). Further, implicit learning ability is resilient to deficits resulting from aging or injury (Verneau et al., 2014; Kal et al., 2016) and is unrelated to intelligence (Maybery et al., 1995). For these reasons, structuring motor practice to bias learning toward implicit learning mechanisms may be effective for robust motor skill learning in many applications, but particularly in the context of skilled sport training and rehabilitation and in individuals who have diminished explicit learning abilities related to aging or brain injury. The effect of social comparative feedback on implicit motor sequence learning and expectancies remains unclear.

Another benefit of the STT task is that it can allow for tracking of spatial and temporal aspects of performance across motor practice. With practice of an STT, improvements in overall performance can be achieved through changes in both spatial (i.e., hand path distance) and temporal (i.e., peak velocity) components of movement (Baird and Stewart, 2018). These variables could be sensitive to social comparative feedback; however, they have not been investigated in previous studies. In particular, peak velocity may provide insight into the learner’s motivation toward the task. Evidence from animals and humans suggests that, when a reward or positive outcome is possible, animals and humans will move with greater speed to achieve it (Seideman et al., 2018; Summerside et al., 2018). In this way, the control of movements may reflect our valuation of subjective goodness or “utility” of an option, where higher speed reflects higher valuation (Shadmehr et al., 2019). The STT provides an opportunity to examine the effect of positive social comparative feedback on the spatial and temporal aspects of motor performance during practice within the context of implicit motor sequence learning.

Therefore, the purpose of this study was to determine the effects of positive social comparative feedback on the learning of a joystick-based implicit motor sequence task and related performance expectancies. It was hypothesized that the group who received positive social comparative feedback would show greater improvements in performance (faster response times) and greater increases in task-related confidence (task specific self-efficacy and perceived competence) reflecting enhanced expectancies, at retention testing than groups that did not receive positive social comparative feedback. A secondary aim of this study was to determine the effect of social comparative feedback on spatial and temporal components of motor performance, such as hand path distance and peak velocity, in order to better characterize how performance changed over practice as a result of feedback type.

## 2. Materials and methods

### 2.1. Participants

Fifty-four non-disabled adults between age 18 and 40 years were recruited from the university and local community. Individuals were included in the study if they were right-hand dominant as measured by the Edinburgh Handedness Questionnaire (Oldfield, 1971) and denied pain or other limitation affecting their ability to move their right arm and hand. Since dopamine plays a major role in learning and motivation (Wise, 2004), individuals were excluded if they were taking medication that impacts dopamine transmission (e.g., dopamine reuptake inhibitors) or were diagnosed by a physician with a disorder affecting dopamine transmission (e.g., Parkinson’s disease). Five individuals were on medications that might impact dopamine transmission and one individual did not return for Session 2; these individuals were not included, leaving 48 participants for final data analysis. This study was powered to find effects at retention. For a repeated measures ANOVA design with within and between group interactions and assuming moderate effect, a total sample size of 42 (3 groups of 14) would provide 80% power to find an effect with two measurements, one at baseline and one at retention (*f* = 0.25, alpha = 0.05, 1-beta = 0.80; number of measurements = 2; corr among rep measures = 0.5; nonsphericity correction = 1; G*Power 3.9.1.2). To account for the possible loss of data, 48 participants (16 per group) was the target recruitment number. All participants provided written informed consent prior to enrollment in the study. The university’s Institutional Review Board approved all procedures. Participants were provided a $10 cash card at the end of each session.

### 2.2. Experimental design

**Figure 1:**
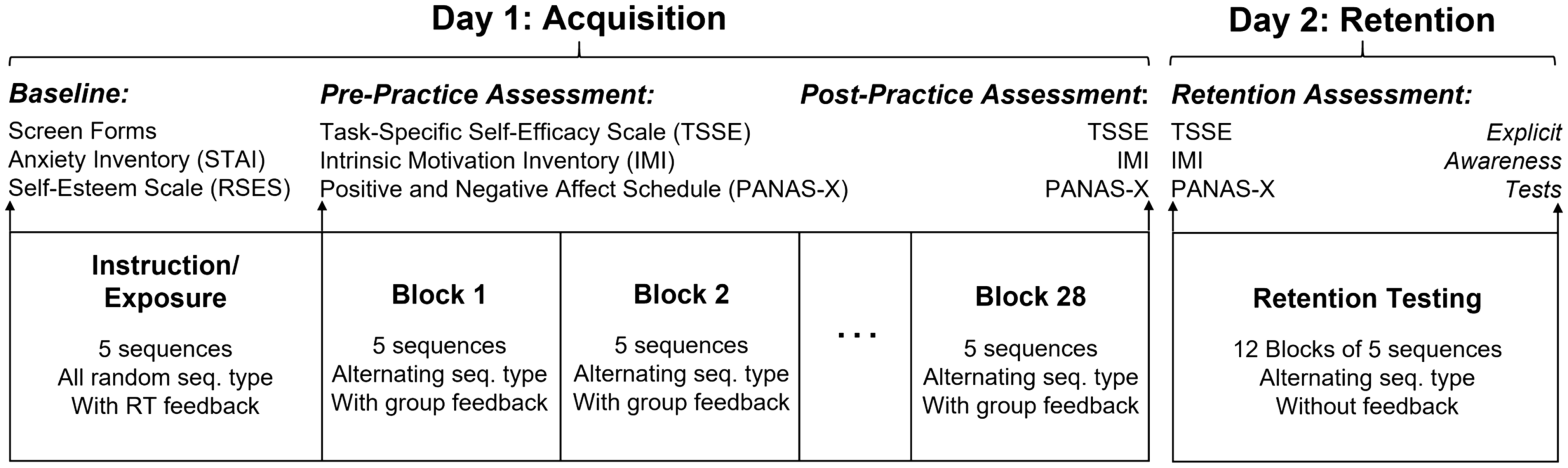
Overview of experimental design.

Participants completed two experimental sessions on consecutive days (Day 1 and Day 2; Figure 1). In each session, participants completed a series of questionnaires and practiced a serial target task (STT) with the right arm and hand. Participants were block randomized into one of three experimental feedback conditions, such that each experimental group had equal numbers of males and females. The three experimental groups received different feedback about their task performance and included a control group (CONTROL), a response time feedback group (RT ONLY), and a response time plus positive feedback group (RT + POS). Previous literature on positive social comparative feedback included groups comparable to the RT ONLY and RT + POS group, but not a group comparable to the CONTROL group (Lewthwaite and Wulf, 2010; Ávila et al., 2012; Chua et al., 2018). We included a CONTROL group to mirror traditional implicit sequence learning task paradigms, where performance feedback is generally not provided (Nissen and Bullemer, 1987). On Day 1, participants completed acquisition practice of the motor task. On Day 2, participants completed retention testing of the motor task and were tested on their explicit awareness of the repeated sequence.

### 2.3. Serial target task

**Figure 2:**
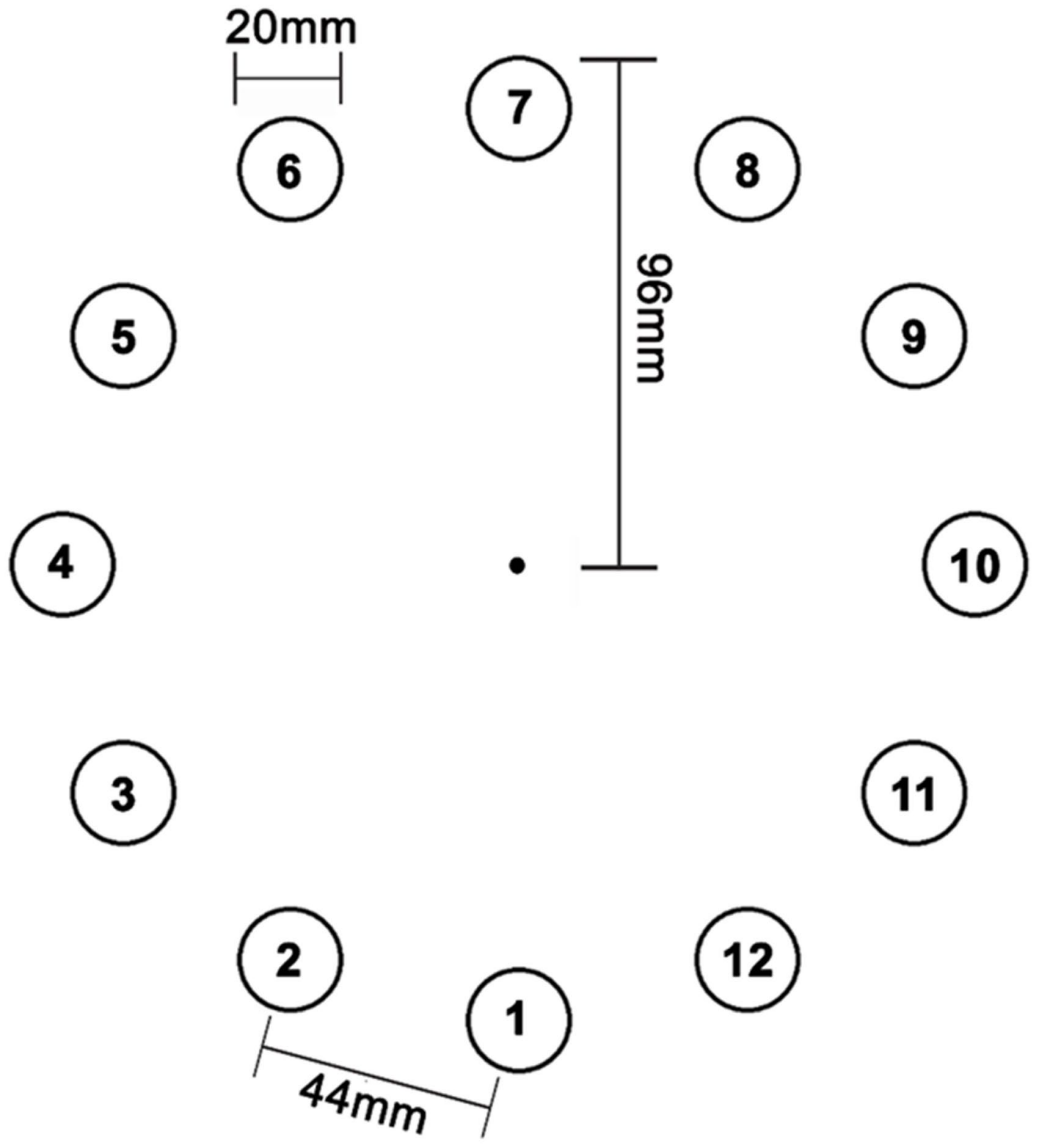
Schematic of the spatial locations of the 12 targets. Each target was 20 mm in diameter with a tangential distance of 44 mm between any adjacent targets. The radius of the circular array was 96 mm. The repeated sequence consisted of targets 11, 10, 5, 9, 7, 3, 6, and 12.

The serial target task (STT) was modified from previous studies (Mang et al., 2014; Baird and Stewart, 2018) such that the present task allowed the joystick to act as the cursor, had no central target, and had a greater number of potential target positions. The central target was removed since the spring in the joystick automatically positions the joystick to center which would not require a goal-directed movement for target capture. Participants sat facing a laptop where the STT was displayed on the screen and held a joystick with the right, dominant hand. Participants used their right arm and hand to move the joystick, which moved a pointer-shaped cursor on the screen in proportion to the joystick movement. Circular targets (20-millimeter diameter) appeared one at a time in one of 12 distinct locations (Figure 2). Before beginning task practice, participants were provided verbal and written instructions about the task goal (i.e., to hit the target as fast as possible). The task required the participant to move the joystick “cursor” to the center of each target until it disappeared, and the next target would appear. The target was considered “hit” and disappeared when the position of the cursor was within 7 millimeters of the center of the target for 500 milliseconds. Targets were presented in alternating random and repeated 8-target sequences; the random sequences were included to help ensure the repeated sequence remained implicit. In addition, the two sequence types allowed for distinction between changes in performance related to more general sensorimotor learning (random sequences) and changes related to sequence-specific motor learning (repeated sequences).

The two sequence types were matched for difficulty based on Fitts’ Law, which considers the distance between the targets and the diameter of the target (Fitts and Peterson, 1964). In this task, the target diameter was the same for all targets; therefore, the repeated sequence and all random sequences were matched on their total straight-line inter-target distance. Individual movements between two targets were assigned a difficulty ID based on Fitts’ Law (difficulty IDs = 2.14, 3.10, 3.61, 3.90, 4.05, and 4.10) where higher ID numbers indicate greater difficulty and longer inter-target distance. The Fitts’ ID numbers were then assigned a rank order for simplicity (e.g., target pairs with ID 2.14 = 1, target pairs with ID 3.10 = 2, etc). The repeated sequence contained one target each at IDs 1, 2, 5, and 6 and two targets each at IDs 3 and 4 (repeated sequence: 11-10-5-9-7-3-6-12). Each random sequence contained the same number of targets at each ID level as the repeated sequence. Participants were not made aware of the presence of any sequences.

Participants completed the STT in blocks that contained 41 targets (a “start” target plus five 8-target sequences per block) and sequence types alternated within a block. Movement to the first target was not included in data analysis, as this target served to initiate movement away from the joystick’s automatic center before beginning the sequenced movements. At the beginning of Day 1, participants completed an exposure block that included 5 random sequences but no feedback to ensure understanding of the task and to provide a baseline measure of performance. After the exposure block, participants completed 28 blocks of practice for a total of 70 repeated sequence repetitions and 70 random sequence repetitions. Group-specific feedback (see below) was provided in written format on the laptop screen after each of the 28 blocks practice. Participants returned for the second session on the following day (Day 2), which measured retention performance (i.e., motor learning) of the STT, and completed 12 blocks of motor practice. These blocks of retention testing were structured the same as the Day 1 practice blocks but did not include any feedback.

Position data from joystick was collected at a rate of 60 Hz using E-Prime 2.0 (Psychology Software Tools, Inc., Sharpsburg, PA). Position data was used for calculation of response time, path distance, and peak velocity. The primary measure of STT performance was response time (time to complete one 8-target sequence). Path distance, a spatial measure of performance, was defined as the total distance traveled to complete all 8 targets in a sequence, where shorter distances indicated straighter hand paths. Peak velocity, a temporal measure of performance, was defined as the peak of velocity for each movement between two targets which was then averaged across all movements in each 8-target sequence. Performance data were separated by sequence type (repeated or random) and averaged across five trials of the same sequence type for statistical analysis.

### 2.4. Feedback

During practice on Day 1, all participants received feedback after each block of five sequences. The control group (CONTROL) received feedback that they completed the block (i.e., “You have completed the block. Take a rest”). The response time only group (RT ONLY) received feedback on their response time to complete all of the targets in the block (i.e., “You completed this block in 86.1 s”) where the feedback provided their actual response time to “hit” all 41 targets in the block. The response time plus positive feedback group (RT + POS) received feedback about their response time with the additional information that their response time was faster than others (i.e., “Your response time was 86.1 s. You were 17.2 s faster than the average”). The social comparative difference (i.e., “You were 17.2 s faster than the average”) was a set percentage of the individual participant’s response time on that block (Wulf et al., 2014). The percentage varied between 14 and 20% for 24 blocks and was reduced to 5% for four randomly selected blocks. Feedback was provided after each of the 28 blocks, where each block contained five sequences of alternating sequence type. Feedback was provided based on the amount of time to complete all of the targets in the block, not related to individual sequence performance, since participants were not made aware of the presence of the sequences during practice.

### 2.5. Explicit awareness testing

At the end of Session 2, participants were tested to evaluate explicit awareness of the repeated sequence pattern. The aim of this testing was to confirm that the task paradigm engaged implicit sequence learning processes without promoting explicit knowledge of the sequence order. Subjective awareness was determined by asking participants if they noticed anything about the motor task. Subjective awareness was defined as the ability to explicitly state that there was a pattern or repeated combinations in the targets. Participants with subjective awareness were tested on their recall awareness whereby the participant was asked to reproduce the sequence by tracing the repeated pattern on a printed paper with the 12-target layout (Figure 2). Participants who did not report subjective awareness where not tested on recall awareness.

All participants were then tested for recognition awareness of the repeated sequence. Participants were first informed of the presence of a repeating pattern and then asked to complete six recognition tests. Each test required participants to view three 8-target sequences play on the laptop, where targets were displayed one at a time. At the end of each test, the participants were asked whether the repeated sequence was present at the “beginning,” “middle,” “end,” or “not at all.” Recognition awareness was defined as the ability to correctly identify two out three positive tests and correctly reject two out three negative tests (Baird and Stewart, 2018).

### 2.6. Surveys and questionnaires

All surveys were collected by subject direct input into RedCap. Prior to STT practice on Day 1, participants completed the Rosenberg Self-Esteem Scale (RSES) (Rosenberg, 1965) and the State Trait Anxiety Index (STAI) (Spielberger et al., 1983; Thomas and Cassady, 2021) to provide additional information about baseline self-esteem and anxiety, respectively. To assess changes in psychosocial factors over practice, participants completed surveys which measured task-specific self-efficacy (Task-Specific Self Efficacy Scale), perceived competence in task performance and interest/enjoyment in the task (subscales of the Intrinsic Motivation Inventory), and general positive affect (Positive and Negative Affect Scale) before and after practice on Day 1 and before retention testing on Day 2.

After the exposure block, participants completed three surveys. The Task-Specific Self-Efficacy Scale (TSSE) measured participants’ self-efficacy related to STT performance (Bandura, 2006; Saemi et al., 2012). The scale asked participants to rate their perceived ability to perform the task on a scale from 0 (“cannot do it at all”) to 100 (“completely certain I can do it”) in intervals of 10 s response times (e.g., How confident are you in your ability to complete the task in 100–109 s?). The scale contained 10 items at 10-s intervals, and ratings were summed to create an overall self-efficacy score for analyses with higher scores indicating higher self-efficacy with a maximum score of 1,000.

A modified version of the Intrinsic Motivation Inventory (IMI) was utilized to survey perceived competence and task interest/enjoyment (Ryan, 1982; Deci and Ryan, 1985). The modified version was adapted for task evaluation and contains four subscales for interest/enjoyment, perceived competence, perceived choice, and pressure/tension. Only the perceived competence and interest/enjoyment subscales were measured due to their relationship to intrinsic motivation which was expected to be sensitive to positive social comparative feedback (Wulf and Lewthwaite, 2016). The perceived competence subscale contains 5 items, each rated on a scale from 1 to 7, and is theorized to be a positive predictor of intrinsic motivation. The interest/enjoyment subscale contains 7 items, each rated on a scale from 1 to 7, and is considered a measure of intrinsic motivation. The items in each subscale were summed where higher scores indicate higher levels of perceived competence or interest/enjoyment. The items in each subscale were summed where higher scores indicate higher levels of perceived competence or interest/enjoyment. For the modified version of the IMI used in the current study, internal consistency, measured by Cronbach’s-⍺, was good for the perceived competence subscale (0.91–0.92) and the interest/enjoyment subscale (0.79–0.87).

The Positive and Negative Affect Scale (PANAS-X) was used to measure positive affect (Watson et al., 1988). This assessment tool is composed of words (e.g., cheerful) or phrases (e.g., dissatisfied with self) that describe different feelings or emotions. Participants were asked to indicate the extent to which they felt this way at the current time on a scale where 1 = very slightly or not at all, 2 = a little, 3 = moderately, 4 = quite a bit, and 5 = extremely. The General Positive Affect subscore was the primary score of interest with a maximum score of 50 (higher scores indicate higher positive affect).

### 2.7. Data analysis

All analyses were conducted in SPSS version 27 (IBM Corp., Armonk, NY). Normality was assessed by Shapiro–Wilk test and visual inspection of histograms. Reciprocal transformations were applied to non-normal data before statistical analysis. To examine differences between groups at baseline, a one-way analysis of variance (ANOVA) was run on age, state anxiety scores, trait anxiety scores, self-esteem scores, baseline psychosocial measure scores (task-specific self-efficacy, perceived competence, interest/enjoyment, positive affect) and baseline performance measures from the exposure block (response time, path distance, and peak velocity). To confirm that sequence-specific learning was present, a repeated measures ANOVA with within-subject factors for block (baseline exposure block; Day 2 middle Block 4) and sequence (random and repeated) and a between-subject factor for group (CONTROL, RT ONLY, RT + POS) was run with response time as the dependent variable. All further analyses were conducted using the repeated sequence data only since the repeated sequence performance reflects implicit sequence learning.

As the primary motor learning analysis, a repeated measures ANOVA with a within-subject factor for block (baseline exposure block; middle block 4 Day 2) and a between-subject factor for group (CONTROL, RT ONLY, RT + POS) was used for the primary variable (response time) and the secondary performance variables (path distance, peak velocity). This middle block of retention performance was selected to represent retention ability while minimizing the effects of warmup and online learning (Bacelar et al., 2022). To explore motor skill acquisition over Day 1, a repeated measures ANOVA with a within-subject factor for block (baseline exposure to the last block of Day 1) and a between-subject factors for group (CONTROL, RT ONLY, RT + POS) was used for all performance variables.

A repeated measures ANOVA with between-subject factors for group (CONTROL, RT ONLY, RT + POS) and a within-subject variable for time (pre-practice, post-practice, and retention) was used to examine task-specific self-efficacy, perceived competence, interest/enjoyment, and general positive affect. The primary outcomes for performance expectancies were perceived competence and task-specific self-efficacy. For all analyses, significance was set at *p* < 0.05. Greenhouse–Geisser correction was utilized when the assumption of sphericity was violated. *Post hoc* analyses were performed to further assess any significant effects. Main effects of group were followed with a Tukey’s HSD to determine the location of differences between groups. Significant group by time interactions were followed with a repeated measures ANOVA separately for each group; paired comparisons between groups at each timepoint were also assessed. Partial eta squared (*η*_p_^2^) estimated the effect size where a value 0.01–0.059 indicates a small effect, 0.06–0.139 indicates a medium effect, and ≥ 0.14 indicates a large effect (Cohen, 1988).

## 3. Results

### 3.1. Participants

Participants were on average 25.4 ± 5.2 years old with 11 females and 5 males in each group. There was no significant difference between groups in age, Rosenberg Self-Esteem scores, or State Trait Anxiety Scale scores (Table 1). In addition, there was no significant difference between groups in any psychosocial variable at baseline (pre-practice scores for task-specific self-efficacy, perceived competence, interest/enjoyment, and positive affect; Table 2) or in any performance variable at baseline (response time, path distance, and peak velocity) as measured during the exposure block (Table 1).

**Table 1:** Group demographics and baseline characteristics.

	Group 1	Group 2	Group 3
	CONTROL	RT ONLY	RT + POS
***n***	16	16	16
**Sex**	11F/5M	11F/5M	11F/5M
**Age (years)**	24.8 (3.7)	26.8 (6.1)	24.8 (5.6)
**RSE score**	35.1 (4.1)	35.0 (3.1)	35.4 (4.1)
**State anxiety score**	33.1 (6.3)	33.1 (6.5)	32.8 (8.8)
**Trait anxiety score**	29.5 (3.7)	27.9 (6.8)	28.8 (6.2)
**Response time (s)**	13.8 (1.4)	14.3 (0.7)	13.7 (1.3)
**Path distance (cm)**	152.0 (12.4)	154.2 (11.4)	154.9 (16.3)
**Peak velocity (cm/s)**	55.0 (7.6)	51.0 (3.7)	56.2 (8.0)
**Feedback**	“You completed the block. Take a rest.”	“You completed the block in 87.2 s.”	“You completed the block in 87.2 s. Your time was 17.5 s faster than the average of others on this block.”

Mean values (standard deviation); F, Female; M, Male; y, years; RSE Score, Rosenberg Self-Esteem Score; no significant differences between groups on any measure.

**Table 2:** Scores from expectancy measures across practice and at retention.

		CONTROL	RT ONLY	RT+POS
**Task-specific Self-efficacy** ^*^	Pre	484.0 (85.0)	477.5 (74.4)	520.0 (125.9)
	Post	568.7 (98.2)^**^	574.4 (86.4)^**^	582.5 (77.4)
	Ret.	555.5 (111.3)^**^	586.2 (76.7)^**^	585.6 (71.4)^**^
**Perceived competence** ^**†**^	Pre	18.6 (6.3)	18.6 (4.8)	20.3 (5.6)
	Post	22.1 (6.3)^**^	20.1 (3.3)	26.3 (5.1)^**^
	Ret.	23.2 (5.5)^**,‡^	20.4 (3.5)	26.0 (4.9)^**^
**Interest/enjoyment**	Pre	24.6 (7.2)	23.5 (6.3)	26.1 (6.6)
	Post	25.9 (8.3)	23.5 (7.1)	26.8 (6.2)
	Ret.	26.1 (8.1)	22.6 (7.4)	26.1 (7.4)
**Positive affect**	Pre	31.7 (15.5)	28.8 (5.6)	33.1 (8.9)
	Post	30.8 (8.4)	29.8 (8.2)	31.5 (9.4)
	Ret.	29.9 (7.6)	28.3 (9.0)	30.6 (11.3)

Mean values (standard deviation) for each time point by group; Ret. = Retention. *Significant main effect of time (*p* < 0.05) on the psychosocial measure, no significant main effect of group or group by time interaction. †Significant group by time interaction (*p* < 0.05). **Significantly different from pre-practice score at *p* < 0.05. ‡Significantly different from post-practice score at *p* < 0.05.

### 3.2. Motor task performance and learning

#### 3.2.1. Sequence-specific effect and acquisition

**Figure 3:**
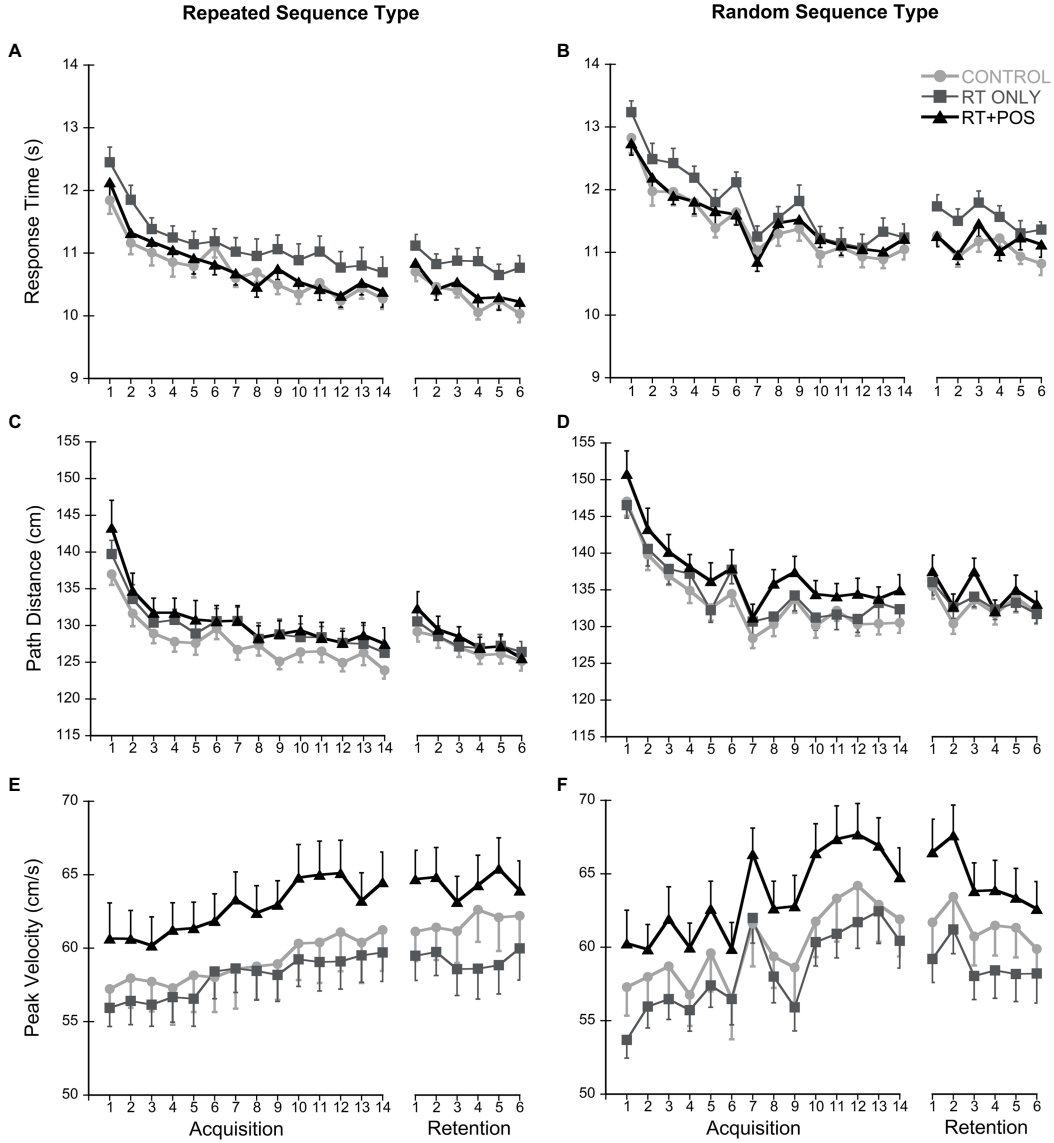
Motor performance variables during Acquisition and Retention. Each data point is the average ± standard error of 5 sequence trials. **(A)** Response time for the repeated sequence; **(B)** Response time for the random sequence; **(C)** Path distance for the repeated sequence; **(D)** Path distance for the random sequence; **(E)** Peak velocity for the repeated sequence; **(F)** Peak velocity for the random sequence.

Sequence specific learning was present as expected; response times were lower for the repeated sequence compared to the random sequence (Figure 3; main effect of sequence (*F*_(1,45)_ = 121.67, *p* < 0.001, *η*_p_^2^ = 0.73). Response times decreased from the start of Day 1 to the middle of Day 2 (main effect of time *F*_(1,45)_ = 584.93, *p* < 0.001, *η*_p_^2^ = 0.93). Similar to the primary retention analysis below (Section 3.2.2 Retention), the type feedback participants received impacted response times (main effect of group, *F*_(1,45)_ = 4.07, *p* = 0.24, *η*_p_^2^ = 0.15), but there was no group by sequence interaction *F*_(1,45)_ = 2.96, *p* = 0.062, *η*_p_^2^ = 0.12).

For acquisition performance of the repeated sequence, response time decreased over task practice on Day 1 (Figure 3A; main effect of time *F*_(7.86,353.85)_ = 127.14, *p* < 0.001, *η*_p_^2^ = 0.74) demonstrating improved task performance across groups. The type of feedback participants received did not impact response times (main effect of group *F*_(2,45)_ = 1.97, *p* = 0.152, *η*_p_^2^ = 0.08; group by block interaction *F*_(15.73,353.85)_ = 0.78, *p* = 0.706, *η*_p_^2^ = 0.03). Path distance also decreased over practice on Day 1 (Figure 3C; main effect of time *F*_(5.17,232.44)_ = 111.17, *p* < 0.001, *η*_p_^2^ = 0.712). The type of feedback participants received did not impact path distance (main effect of group *F*_(2,45)_ = 0.91, *p* = 0.411, *η*_p_^2^ = 0.04; group by block interaction *F*_(10.33,232.44)_ = 0.44, *p* = 0.931, *η*_p_^2^ = 0.02). Finally, peak velocity increased over practice on Day 1 (Figure 3E; main effect of time *F*_(4.19,188.42)_ = 16.93, *p* < 0.001, *η*_p_^2^ = 0.27). The type of feedback participants received did not impact peak velocity (main effect of group *F*_(2,45)_ = 1.75, *p* = 0.186, *η*_p_^2^ = 0.07; group by block interaction *F*_(8.38,188.42)_ = 0.75, *p* = 0.653, *η*_p_^2^ = 0.32).

#### 3.2.2. Motor learning: Retention

For retention performance of the repeated sequence on Day 2, response times decreased from baseline (Figure 3A; main effect of time *F*_(1,45)_ = 635.63, *p* < 0.001, *η*_p_^2^ = 0.93) demonstrating implicit learning across groups. The type of feedback participants received impacted response times (main effect of group *F*_(2,45)_ = 4.442, *p* = 0.017, *η*_p_^2^ = 0.17), where the CONTROL group showed lower response times than the RT ONLY group (*p* = 0.024). No other pairwise comparisons were significant, and the group by block interaction was not significant (*F*_(2,45)_ = 1.14, *p* = 0.328, *η*_p_^2^ = 0.05). Additionally, path distance got shorter from baseline to the middle of Day 2 (Figure 3C; main effect of time *F*_(1,45)_ = 347.11, *p* < 0.001, *η*_p_^2^ = 0.89). However, the type of feedback participants received did not impact path distance (main effect of group *F*_(2,45)_ = 0.20, *p* = 0.820, *η*_p_^2^ = 0.01; group by block interaction *F*_(2,45)_ = 0.94, *p* = 0.910, *η*_p_^2^ < 0.01). Finally, peak velocity increased from baseline to the middle of Day 2 (Figure 3E; main effect of time *F*_(1,45)_ = 55.63; *p* < 0.001, *η*_p_^2^ = 0.55). The type of feedback participants received impacted peak velocity (main effect of group *F*_(2,45)_ = 3.25, *p* = 0.58, *η*_p_^2^ = 0.13), where the RT + POS group showed higher peak velocities than the RT ONLY group (*p* = 0.046). Peak velocity in the CONTROL group was not significantly different from either of the other two groups. The group by block interaction was not significant (*F*_(2,45)_ = 0.01, *p* = 0.991, *η*_p_^2^ < 0.01).

### 3.3. Explicit awareness

Ten participants reported recognizing a pattern during practice (subjective awareness), however, seven of the ten participants could not reproduce any part of the repeated sequence. Two participants from the CONTROL group and one participant from the RT + POS were able to reproduce part of the repeated sequence in the correct sequential order. None were able to reproduce the whole sequence. Nine participants were identified as having recognition awareness of the repeated sequence (two from CONTROL, three from RT ONLY, and four from RT + POS). Only two participants had recognition awareness and were able to recall part of the repeated sequence in the correct sequential order (one each from CONTROL and RT + POS group). The similar distribution of explicit awareness across groups suggests that the provision of performance related feedback did not impact explicit awareness of the repeated sequence.

### 3.4. Performance expectancies

**Figure 4:**
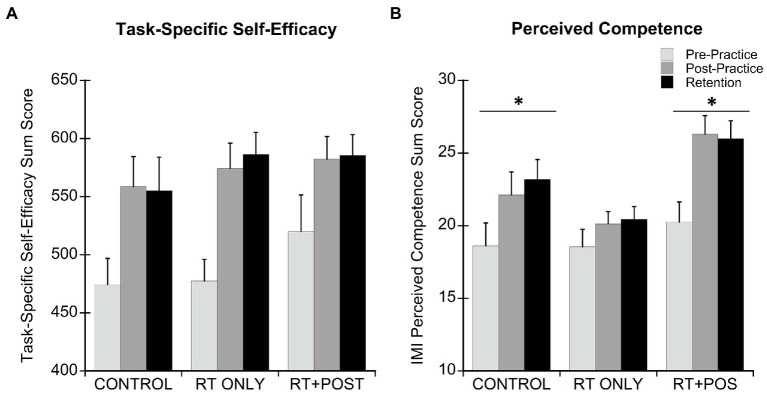
**(A)** Task-Specific Self-Efficacy Scores ± standard deviation for each group at pre-practice, post-practice, and retention**. (B)** Intrinsic Motivation Inventory (IMI) Perceived Competence subscale sum scores ± standard error for each group at pre-practice, post-practice, and retention. Maximum scores for the IMI Perceived Competence subscale is 35. *Significant main effect of time within group.

Task-Specific Self-Efficacy scores for each group were assessed at pre-practice, post-practice and retention (Table 2; Figure 4). One participant from the CONTROL group was missing retention data for the TSSE due to a technical difficulty. This person was dropped from this analysis only. As expected, task-specific self-efficacy scores increased with task practice (main effect of time *F*_(1.35,59.59)_ = 28.18, *p* < 0.001, *η*_p_^2^ = 0.39). *Post hoc* paired *t*-tests between time points (pre to post, post to retention, and pre to retention) revealed that, for all groups, TSSE scores increased from pre-practice to retention (CONTROL *p* = 0.013; RT ONLY *p* < 0.001; RT + POS *p* = 0.017). Only the CONTROL and RT ONLY groups showed increases in TSSE scores from pre to post on Day 1 (CONTROL *p* = 0.010; RT ONLY *p* < 0.001; RT + POS *p* = 0.069). For all groups, scores were maintained at retention and changes that occurred between post and retention were not significant (CONTROL *p* = 0.416: RT ONLY *p* = 0.240; RT + POS *p* = 0.818). The type of feedback provided did not impact task-specific self-efficacy ratings (no main effect of group, *p* = 0.62; no group by time interaction, *p* = 0.528).

Perceived competence was assessed for each group at pre-practice, post-practice and retention (Table 2; Figure 4B). The groups’ perceived competence scores were not different at pre-practice (*p* = 0.628), but changed differently over time, as indicated by a significant group by time interaction (*F*_(2.75,61.76)_ = 4.21; *p* = 0.011, *η*_p_^2^ = 0.16). The CONTROL group (main effect of time *F*_(1.48,22.25)_ = 19.42, *p* < 0.001, *η*_p_^2^ = 0.56) and RT + POS (main effect of time *F*_(1.48,22.25)_ = 29.89, *p* < 0.001, *η*_p_^2^ = 0.67) group showed a significant increase in perceived competence over time while the RT ONLY group did not (no main effect of time, *p* = 0.121). The CONTROL group’s perceived competence scores changed by 3.5 ± 3.4 points from pre-practice to post-practice (*p* < 0.001) and 1.1 ± 2.0 from post-practice to retention (*p* = 0.049). The RT + POS group’s perceived competence scores changed by 6.1 ± 4.0 from pre-practice to post-practice (*p* < 0.001) and −0.31 ± 2.1 from post-practice to retention (*p* = 0.558). Comparing between group at each timepoint, the RT + POS group showed significantly higher perceived competence scores than the RT ONLY group at post-practice and retention (*p* < 0.01). No other pairwise comparisons between groups at each timepoint were significant.

The type of feedback provided had no impact on positive affect, as measured with the General Positive Affect score from the PANAS-X, or task interest/enjoyment, as measured with the interest/enjoyment subscale of the IMI (Table 2). Interest/enjoyment (no main effect of time, *p* = 0.463) and positive affect (no main effect of time, *p* = 0.071) did not change over time.

## 4. Discussion

This study examined the effect of positive social comparative feedback on the learning of a joystick-based motor sequence task that allowed for the examination of implicit sequence learning. As expected, and regardless of feedback group, overall performance on the task improved over practice and at retention as seen by faster response times. The expected sequence-specific effect, where learners were faster at performing the repeated sequence than the random sequence, was also present signifying that implicit sequence learning occurred. However, the primary hypothesis, which was that the group who received positive social comparative feedback (RT + POS) would show greater improvements in performance and greater increases in task-related confidence than groups that did not receive this feedback, was not confirmed in the current study. There was no difference between the feedback groups on response time during acquisition and the CONTROL group had faster response times than the RT ONLY group on retention; no other between group differences on response time were identified. Expectancies as measured by perceived competence scores increased over practice in the CONTROL and RT + POS group, but not for the RT ONLY group. The RT + POS group showed higher peak velocities than the other groups during acquisition and at retention, a potential behavioral reflection of higher intrinsic motivation in the RT + POS group. Overall, these results showed that RT + POS feedback did not promote motor skill acquisition or learning more than the other feedback types, and RT + POS was not superior to CONTROL feedback for enhancing expectancies as measured by self-report surveys.

Our results are in conflict with several previous studies that found that performance feedback with positive social comparison benefits learning more than performance feedback without social comparison (Lewthwaite and Wulf, 2010; Wulf et al., 2012, 2014; Pascua et al., 2015). Importantly, these studies did not include a group comparable to our no feedback CONTROL group. Traditional paradigms aimed at assessing implicit sequence learning do not provide post-response feedback during practice (Reber, 1989). We included a no feedback group in order to make comparisons to this traditional approach known to support this form of learning. By including both the no feedback control group and the response time only group, the current results were designed to build on the evolving knowledge within both sets of literature (implicit learning and OPTIMAL-based studies). Overall, our findings indicate that there may be specific motor learning mechanisms, like implicit learning, where expectancy enhancing feedback is not necessarily better for learning or confidence than no feedback. Future research using different task paradigms that include a no feedback group is warranted to replicate our findings and to determine if other tools for enhancing expectancies besides positive social comparative feedback might be more effective in tasks that rely on implicit learning processes.

Response time feedback without context (RT ONLY) appeared to be detrimental for motor sequence learning compared to no feedback (CONTROL) and positive social comparative feedback (RT + POS). A possible explanation for this effect is that the RT ONLY group was given performance information without an easily accessible means for assessing whether their performance was good or bad. Knowledge of results feedback, such as response time, encourages learning through cognitive processes rather than conditioning responses (Salmoni et al., 1984; Maier et al., 2019). Therefore, response time feedback during an implicit sequence learning task may not be helpful to the learner without easily accessible context for evaluating the performance information. According to the OPTIMAL theory, feedback that enhances expectancies helps the learner to reduce focus on the self and increase focus on the task goal, which then supports motor skill acquisition and learning (Wulf and Lewthwaite, 2016). Feedback about performance without immediate context might result in increased internal focus, whereby the learner is attending to their response time and attempting to compare the current response time to response times from previous blocks. This idea is supported in the perceived competence data. The RT ONLY group did not show an increase in perceived confidence despite the fact that their performance improved over time. This disconnect between actual performance improvements and perceptions of competence could be attributed to increased self-focus on their response time with an inability to determine whether they are meaningfully improving performance or not. Future studies should aim to determine how performance feedback without immediate context during novel skill practice influences attentional and cognitive aspects of learning.

The RT + POS and RT ONLY groups achieved similar response times over practice and at retention. However, the underlying components of the movement were different with the RT + POS showing higher peak velocities. Hand path distance and peak velocity are kinematic variables that contribute to the resulting response time (Moisello et al., 2009; Baird and Stewart, 2018). Shorter hand paths indicate greater spatial accuracy along the movement trajectory while higher peak velocities indicate faster reach speeds, both of which lead to reduced response times. A learner may improve their response time by utilizing straighter/shorter hand paths (spatial control pattern), higher peak velocities (temporal control pattern), or a combination of both. Prior work suggests that changing motor practice conditions, such as the intensity of cardiovascular exercise that occurs before a practice session, can alter whether the learner utilizes a spatial or temporal approach to improve performance (Baird et al., 2018). Our results suggest that feedback content may influence a learner’s approach to improving their performance since the RT + POS group showed higher peak velocities than the RT ONLY group. Peak velocity may provide additional information about the learner’s motivation toward the task. This is supported by the idea that movement vigor, including movement velocity, reflects the learner’s valuation of the expected outcome (Shadmehr et al., 2019). For example, in monkeys, the peak velocity of eye movements (saccades) increased as the probability of reward increased (Seideman et al., 2018). In humans, reaches were faster toward rewarded versus nonrewarded targets (Summerside et al., 2018). Taken together, this suggests that, when a reward is possible, animals will move with greater speed to achieve it. In this way, the control of movements may reflect our valuation of subjective goodness or “utility” of an option, where higher speed reflects higher valuation (Shadmehr et al., 2019). The higher peak velocities in the RT + POS group reflects the learners’ higher subjective value of the task when positive social comparison was provided in addition to response time. This finding suggests that speed might be an important variable to consider when studying motor skill learning, enhanced expectancies, and intrinsic motivation. It is also possible that the higher peak velocity in the RT + POS group reflect the focus of the feedback itself. According to the OPTIMAL theory, positive social comparative feedback aligns the learner’s actions to the task goal (Wulf and Lewthwaite, 2016). The task goal in the current study was to move fast and feedback was provided based on response times, including the social comparative feedback. Future work should aim to determine if positive social comparative feedback consistently leads to changes in the component of performance that is stated as the task goal and the focus of the feedback (e.g., speed versus spatial) or if this type of feedback encourages a temporal approach to improving performance.

The results of the current study suggest that positive social comparative feedback was not effective in enhancing expectancies beyond that which was achieved in a CONTROL no feedback condition. According to the OPTIMAL theory, positive social comparative is a tool to enhance a learners’ expectancies about future performance, thereby increasing motivation toward the task leading to better motor performance and learning. The feedback provided impacted some measures of expectancies (perceived competence) but not others (task-specific self-efficacy, task interest/enjoyment, positive affect). All groups showed gains in task-specific self-efficacy, which was expected given that all groups improved performance over practice and previous performance level predicts self-efficacy (Moritz et al., 2000; Wulf et al., 2014). However, the hypothesized group effect, where the RT + POS would show the greatest gains, was not present. This could be attributable to the TSSE scale, which included a range of response time windows; the time windows selected may not have been optimal for detecting differences between groups. In addition, other expectancy-related measure (task interest/enjoyment and positive affect) were not different by group. The absence of change in task interest and enjoyment has been noted previously (Lewthwaite and Wulf, 2010), and may be explained by a global characteristic of the task practice (i.e., highly repetitive simple lab task) and not the feedback. On the other hand, the feedback provided impacted self-assessments of perceived competence, where the CONTROL and RT + POS group showed increased perceived competence and the RT ONLY group did not. These results suggest no feedback is better for supporting self-assessed perceived competence than performance feedback without context. If providing performance related feedback during practice, which removes the need for historical recollection of performance on a previous block, comparative context may be necessary for supporting perceptions of competence and enhancing expectancies. Ultimately, if the feedback provided in the current study failed to support competence and enhance motivation toward the task, the expected benefit to motor performance and learning would likely not be present. However, the study was not specifically powered to identify differences between groups on expectancy measures, and therefore, may have been underpowered to identify relatively small effects of positive social comparative feedback on expectancies.

The joystick-based motor sequence task allowed for the investigation of the spatial and temporal aspects of motor performance as well as examination of general sensorimotor learning and implicit sequence learning. However, this task does not represent all types of motor skill learning, and our results may be specific to this task paradigm. In addition, positive social comparative feedback is a type of feedback manipulation intended to enhance the learners’ expectancies; however, there are many potential feedback manipulations that might enhance a learners’ expectancies and benefit motor learning (Chiviacowsky et al., 2012; Saemi et al., 2012; Wulf et al., 2012). The feedback approach in the current study was based on prior research (Wulf et al., 2014), but the best parameters for providing expectancy enhancing feedback have not been established. As such, the parameters of the social comparative feedback provided in this study may not have been the most effective approach for enhancing performance and expectancies in the RT + POS group. Other forms of feedback, such as general feedback indicating that the participant was performing better than others without knowledge of results like response time, might be more effective at supporting confidence and implicit learning. The feedback manipulation involved nonveridical comparative values in order to maintain tight control of feedback delivery in a task that lacked age-matched normative values. While this is valid approach for scientific investigation, nonveridical feedback may not be a feasible clinical intervention. In addition, some participants may not have believed the positive social comparative feedback. However, group differences in expectancies and performance were still present despite this. Future studies could include assessment of feedback believability to determine whether believability influences performance or expectancy improvements. Finally, it is possible that the current study was underpowered to identify effects since our power analysis assumed a moderate effect based on previous studies; these prior studies may have overestimated true effect sizes due to general methodological concerns in the field (Bacelar et al., 2022).

## 5. Conclusion

Positive social comparative feedback indicates to the learner that they are performing better than others. Contrary to our hypothesis, this type of feedback did not result in better overall performance (i.e., lower response times) at retention than no feedback or performance feedback without social context. While the response time only and positive social comparative feedback group showed similar response times, the underlying components of the movement were different, where the group that received positive social comparison demonstrated higher peak velocities between targets. Also contrary to our hypothesis, expectancies, as measured by self-report surveys, were enhanced similarly in the no feedback and positive social comparative feedback groups. Response time feedback without social context was detrimental to both learning and expectancies as compared to a no feedback condition. If providing performance related feedback during practice of a skill that relies on implicit sequence learning, comparative context, which removes the need for historical recollection of performance on a previous block, may be necessary to enhance expectancies and support learning.

## Data availability statement

The original contributions presented in the study are included in the article/supplementary material, further inquiries can be directed to the corresponding author.

## Ethics statement

The studies involving human participants were reviewed and approved by the University of South Carolina’s Institutional Review Board. The patients/participants provided their written informed consent to participate in this study.

## Author contributions

AL and JS contributed to conception and design of the study. AL collected data, performed statistical analysis, and wrote the first draft of the manuscript. RB and LJ assisted with data collection. AL, JS, DO, SW, and SF assisted with interpretation of data. All authors contributed to the article and approved the submitted version.

## Funding

This work was supported by the American Heart Association Predoctoral Fellowship (20PRE35180106), and the University of South Carolina’s Presidential Fellowship, and the University of South Carolina’s Office of the Vice President for Research SPARC Graduate Research Grant.

## Conflict of interest

The authors declare that the research was conducted in the absence of any commercial or financial relationships that could be construed as a potential conflict of interest.

## Publisher’s note

All claims expressed in this article are solely those of the authors and do not necessarily represent those of their affiliated organizations, or those of the publisher, the editors and the reviewers. Any product that may be evaluated in this article, or claim that may be made by its manufacturer, is not guaranteed or endorsed by the publisher.
